# Sequence diversity and natural selection at domain I of the apical membrane antigen 1 among Indian *Plasmodium falciparum *populations

**DOI:** 10.1186/1475-2875-6-154

**Published:** 2007-11-22

**Authors:** Sheena Garg, Mohammad T Alam, Manoj K Das, Vas Dev, Ashwani Kumar, Aditya P Dash, Yagya D Sharma

**Affiliations:** 1 Department of Biotechnology, All India Institute of Medical Sciences, Ansari Nagar, New Delhi, India; 2 National Institute of Malaria Research (Field Station), Car Nicobar, Andaman and Nicobar Islands, India; 3 National Institute of Malaria Research (Field Station), Kamrup, Assam, India; 4 National Institute of Malaria Research (Field Station), Goa, India; 5 National Institute of Malaria Research, Delhi, India

## Abstract

**Background:**

The *Plasmodium falciparum *apical membrane antigen 1 (AMA1) is a leading malaria vaccine candidate antigen. The complete AMA1 protein is comprised of three domains where domain I exhibits high sequence polymorphism and is thus named as the hyper-variable region (HVR). The present study describes the extent of genetic polymorphism and natural selection at domain I of the *ama1 *gene among Indian *P. falciparum *isolates.

**Methods:**

The part of the *ama*1 gene covering domain I was PCR amplified and sequenced from 157 *P. falciparum *isolates collected from five different geographical regions of India. Statistical and phylogenetic analyses of the sequences were done using DnaSP ver. 4. 10. 9 and MEGA version 3.0 packages.

**Results:**

A total of 57 AMA1 haplotypes were observed among 157 isolates sequenced. Forty-six of these 57 haplotypes are being reported here for the first time. The parasites collected from the high malaria transmission areas (Assam, Orissa, and Andaman and Nicobar Islands) showed more haplotypes (H) and nucleotide diversity π as compared to low malaria transmission areas (Uttar Pradesh and Goa). The comparison of all five Indian *P. falciparum *subpopulations indicated moderate level of genetic differentiation and limited gene flow (Fixation index ranging from 0.048 to 0.13) between populations. The difference between rates of non-synonymous and synonymous mutations, Tajima's D and McDonald-Kreitman test statistics suggested that the diversity at domain I of the AMA1 antigen is due to positive natural selection. The minimum recombination events were also high indicating the possible role of recombination in generating AMA1 allelic diversity.

**Conclusion:**

The level of genetic diversity and diversifying selection were higher in Assam, Orissa, and Andaman and Nicobar Islands populations as compared to Uttar Pradesh and Goa. The amounts of gene flow among these populations were moderate. The data reported here will be valuable for the development of AMA1-based malaria vaccine.

## Background

The *Plasmodium falciparum *malaria is one of the major causes of morbidity and mortality in tropical and subtropical countries. Worldwide, approximately 300–500 million people are affected each year with the disease and children below 5 years of age and pregnant women are the largest target groups. The global burden of human malaria is continued to increase due to lack of an effective malaria vaccine, development of insecticide resistance in the *Anopheles*, and drug resistance in the parasite. Several parasite molecules have been tested for their potential as a vaccine candidate antigens [1]. Majority of these antigens are expressed on the parasite's surface or the parasitized erythrocytes. The *Plasmodium *proteins expressed on the parasite's surface are more exposed to human immune system and thus have been found to exhibit high antigenic diversity [2]. Genetic polymorphisms in the parasite antigens help them to evade the host's protective immune response but at the same time it hampers the development of an effective vaccine which can contain this disease.

The *P. falciparum *apical membrane antigen 1 (AMA1) is an 83 kDa membrane protein expressed in the late schizont stage of the parasite [3,4]. Although the exact function of AMA1 is not known, its role in merozoite invasion has been documented by several investigators and antibodies raised against this protein have been shown to block invasion of the parasites into human RBCs [5-9]. Natural immune responses (both humoral and cellular) against the AMA1 antigen have been observed in populations exposed to *P. falciparum *malaria [10]. Thus, AMA1 has been included as one of the potential components of an asexual stage multivalent malaria vaccine. An AMA1-based malaria vaccine has recently been tested in phase I clinical trial [11]. Although AMA1 is less polymorphic than other sporozoite or merozoite proteins, some extent of variation are present across the entire AMA1 sequence [12-14] but the rate of non-synonymous (dN) mutations at domain I (aa 138–308) has always been higher indicating that this domain is under strong diversifying selection [13,15,16].

Antigenic variation in *Plasmodium *has hampered malaria vaccine development because the antibodies elicited against one allele of an antigen might not necessarily inhibit RBCs invasion by the parasites expressing different alleles of that antigen [17,18]. Therefore, polymorphism studies on the AMA1 antigen of natural *P. falciparum *populations need to be done before initiating any clinical trials of a vaccine using this antigen. This study describes the extent of antigenic diversity and natural selection at domain I of AMA1 among Indian *P. falciparum *populations. The results obtained here will have significant implications in malaria vaccine development.

## Methods

### Parasite collection

Patients attending malaria clinics at Kamprup (Assam), Cuttack (Orissa), Car Nicobar (A & N), Ghaziabad and Aligarh (UP) and Panjim (Goa) were tested for the presence of *P. falciparum*. About 50–100 μl of blood was collected from the microscopically *P. falciparum *positive patients with their informed consent. The institutional ethical guidelines were followed for blood collection.

### DNA isolation and PCR amplification of the *ama 1 *gene

DNA from *P. falciparum *infected blood was isolated as described previously [19]. This DNA was used to amplify the complete 2 kb *ama*1 gene using the AMA1-F (5'-ACAAAAATGAGAAAATTATACTGC-3') and AMA1-R (5'-TTTTAATAGTATGGTTTTTCCATC-3') primers. The cycling parameters for the primary PCR were as follows: 10 minutes initial denaturation at 94°C followed by 35 cycles with 1 minute denaturation at 94°C, 1 minute annealing at 55°C, 2 minute extension at 72°C and a final 10 minute extension at 72°C. The primary PCR product was diluted 10-times and 2 μl of it was used in nested-PCR to amplify the 500 bp region encompassing domain I (or hyper-variable region-HVR) using the AHVR-F (5'-CTGGAACTCAATATAGACTTC-3') and AHVR-R (5'-TTCTTTCTAGGGCAAACTTTTTC-3') primers. The cycling parameters for nested primers were the same as for primary PCR primers except that the extension at 72°C was carried out for 1 minute.

### Nucleotide sequencing and sequence analysis

The desired 500 bp DNA band was excised from the agarose gel and purified as described earlier [19]. Sequencing of the product was done from both strands using AHVR-F and AHVR-R primers. Sequencing PCR cycling parameters and other downstream protocols for sequencing were same as described earlier [19]. The sequences were edited and the translated amino acids were aligned amongst themselves and also with *P. falciparum *3D7 [GenBank; NC_004315], Dd2 [GenBank; AASM01002002] and HB3 [GenBank; AANS01001407] strains AMA1 sequences using GeneDoc Version 2.6.002 program [20]. In addition, BLAST searches were also performed to compare these AMA1 haplotypes with the other AMA1 sequences available in the GenBank database. The sequences reported in this paper have been deposited in the database [GenBank; EF413088–EF413170].

### Statistical and phylogenetic analyses

The numbers of segregating sites (S), observed nucleotide diversity per site between any two sequences assuming that the sample is random (π), number of haplotypes (H), haplotype diversity (Hd), and average number of pairwise nucleotide differences within population (K), linkage disequilibrium (LD) and recombination parameters (R and Rm) were calculated using DnaSP ver. 4. 10. 9 [21]. The π diversity was also calculated on sliding window of 100 bases, with a step size of 25 bp in order to estimate the step-wise diversity across domain I. The rates of synonymous (dS) and non-synonymous (dN) mutations were computed on MEGA version 3.0 [22] using the method of Nei and Gojobori's [23] with the Jukes and Cantor (JC) correction. The dN-dS difference and Tajima's D [24] test statistics were applied to test the neutral theory of evolution. Positive values for Nei-Gojobori (dN-dS) and Tajima's D correspond to positive natural selection whereas negative values correspond to negative or purifying selection [25]. In addition, McDonald & Kreitman (MK) [26] test was also applied as a test of neutrality taking partial *P. reichenowi *AMA1 sequence as an outgroup using DnaSP. The MK test compares dS and dN mutations within and between species. Under neutrality, the ratio of dN/dS mutations between species should be the same as the ratio of dN/dS mutations within species. However, when the dN/dS ratio is greater between species than within species, it is said to be under positive natural selection. In case of negative selection, the dN/dS ratio between species is lower than within species. The genetic differentiation among the parasite populations was calculated in terms of fixation index (Fst) that estimates diversity within a subpopulation with respect to total genetic diversity. In addition, average number of pairwise nucleotide differences (Kxy), nucleotide substitution per site (Dxy), and net nucleotide substitution per site (Da) between populations were also calculated. The above parameters were also estimated on DnaSP. Phylogenetic analysis was performed by neighbour-joining (NJ) method with Kimura 2-parameter distance matrix [27] in MEGA version 3.0 [22].

## Results

### Haplotype diversity and amino acid changes at domain I

The nucleotide sequence encoding domain I of the AMA1 was determined from 157 clinical isolates collected from five different geographical regions (Assam, n = 28; Orissa, n = 35; A & N, n = 40; UP, n = 36; and Goa, n = 18) of India with different malaria transmission intensities. The analysis of the 456 bp sequence obtained (corresponding to 448–903 bp region of the *ama1 *gene encoding for amino acid residues 150–301) for these 157 isolates revealed that, 418 nucleotide positions were either monomorphic or invariable. Remaining 44 (9.64%) sites were polymorphic (8 singleton variables and 36 parsimony informative), that generated 57 different AMA1 haplotypes (H1 to H57) among Indian *P. falciparum *populations (Additional File 1). The two predominant AMA1 haplotypes were H56 (13.37%, n = 157) and H57 (15.92%, n = 157) (Additional File 1). Thirty-one haplotypes (H1 – H31) were unique as each one of them was found in a single isolate only. The BLAST search against all *P. falciparum *AMA1 sequences available in the GenBank database confirmed that 46 of these 57 haplotypes were new as they have not been reported earlier from any other region of the world. The remaining eleven haplotypes showed 100% identity with the AMA1 sequences reported from other countries [12,13,16,28]. The H56 was the only haplotype that showed 100% identity with the already reported AMA1 sequences [AAG50135] from Indian *P. falciparum *isolate [13]. Furthermore, none of the isolates had identical sequences to the 3D7, Dd2 or HB3 AMA1 alleles.

The number of AMA1 haplotypes in Assam (H = 20), Orissa (H = 23), and A & N (H = 18) were higher than those seen in UP (H = 10) and Goa (H = 7). Majority (14.28%, n = 28) of the isolates from Assam showed H48 haplotype which was not found among the isolates sequenced from the four other areas. Similarly, H57 was the predominant haplotype in Orissa and A & N but not seen in Assam (Additional File 2). Certain haplotypes were found to be region specific, and also none of the 57 haplotypes was common to all the five study areas (Additional File 2). Most (47.22%, n = 36) of the isolates from UP had H56 AMA1 haplotype that was not found in Goa isolates.

Amino acid polymorphisms were observed at 29 of the 152 codons (456 bp) across the entire domain I (Additional File 1). Of these 29 polymorphic sites, 17 [codons-162 (N/K), 167 (T/K), 173 (N/K), 175 (D/Y), 189 (L/P), 190 (M/I), 196 (D/N), 199 (R/K), 206 (K/E), 218 (S/P), 225 (N/I), 228 (N/K), 242 (Y/D), 244 (D/N), 267 (E/Q), 269 (K/I), and 296 (D/H)] showed di-morphic, 8 [codons 172 (G/E/V), 204 (N/D/G), 207 (Y/D/V), 230 (K/E/Q), 243 (N/K/E), 282 (K/I/N), 283 (S/L/P) and 285 (Q/E/R)] showed tri-morphic, 3 [codons 187 (N/E/K/D), 200 (D/L/H/R) and 201 (F/L/S/V)] showed tetra-morphic and only one site [codon 197 (G/D/H/Q/V)] showed penta-morphic alleles.

The number of polymorphic codons and number of total mutations for Assam (25 polymorphic codons and 37 mutations), Orissa (27 polymorphic codons and 38 mutations) and A & N (24 polymorphic codons and 33 mutations) isolates were almost equal but greater than the isolates from UP (20 polymorphic codons and 26 mutations) and Goa (16 polymorphic codons and 20 mutations). Sixteen of the 29 polymorphic codons were common to all the five regions whereas, remaining 13 codons were showing region-specific polymorphisms. The N173K, L189P, and D244N mutations were found among the isolates from Assam and Orissa only but not among A & N, UP and Goa isolates. The codon polymorphisms D175Y, D/N204G, Y/D207V, S218P and Q/E285R were A & N specific whereas G/E172V, E/N/K187D, R199K and K269I were Orissa-specific. Only two Assam-specific mutations were observed (K/I282N and S/L283P) while no region-specific mutations was found among the isolates sequenced from UP and Goa.

### Nucleotide diversity and genetic differentiation

**Figure 1:**
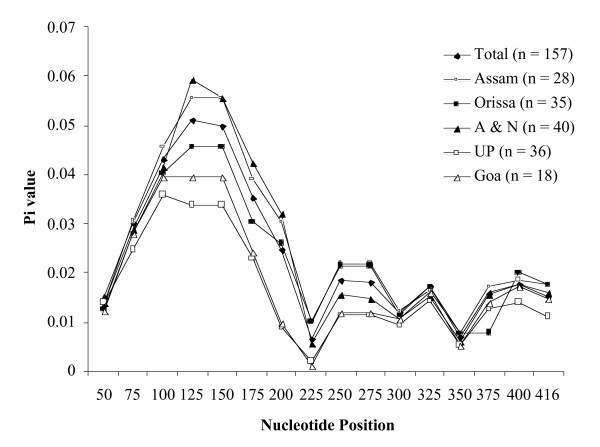
Sliding window plot of the nucleotide diversity per site (π) comparing the level of genetic diversity among the domain I sequences of the parasite from all five study areas. The π values were calculated on DnaSP with window length 100 bp and step size of 25 bp. In all study areas, the maximum diversity was seen between the nucleotide positions 50 and 200 bps. n; number of *P. falciparum *isolates.

The haplotype (gene) diversity (Hd) for all 157 sequences was calculated to be 0.948 ± 0.010 SD. The average number of pairwise nucleotide differences within Indian *P. falciparum *population (K) was 10.13 with the overall π diversity 0.0222 ± 0.0005 SD (Table 1). However, the sliding window analysis of the entire 456 bp region estimated π diversity ranging from 0.00668–0.05126 with the highest value between nucleotide positions 50 and 200 (0.01501 to 0.02488) (Figure 1). A total of 12 minimum number of recombination (Rm) events were observed between adjacent polymorphic sites (Table 2). The estimates of R = 4Nr between adjacent sites and per gene were 0.123 and 56.2 respectively. The haplotype diversity (Hd), nucleotide diversity per site (π) and average number of pairwise nucleotide differences within population (K) showed regional variations (Table 1). The π diversity was highest between nucleotide positions 50 and 200 among all five populations as estimated by sliding window method using a step size of 25 bp (Figure 1).

**Table 1:** Measures of DNA sequence polymorphisms and tests of neutrality at domain I of AMA 1 among Indian *P. falciparum *populations

**Study areas (n = 157)**	**Segregating sites (S)**	**Singleton variable sites**	**Parsimony informative sites**	**Total no. of mutations**	**K**	**H**	**Hd ± S.D**	**π ± S.D**	**dN-dS ± S.E**	**Tajima's D**
Assam (n = 28)	34	6	28	38	10.97	20	0.971 ± 0.018	0.0240 ± 0.0009	0.029 ± 0.006	0.461
Orissa (n = 35)	35	5	30	40	9.96	23	0.948 ± 0.025	0.0218 ± 0.0015	0.027 ± 0.005	0.094
A & N (n = 40)	34	6	28	35	10.34	18	0.926 ± 0.022	0.0226 ± 0.0008	0.027 ± 0.007	0.895
UP (n = 36)	24	6	18	26	7.31	10	0.748 ± 0.066	0.0160 ± 0.0014	0.020 ± 0.005	0.576
Goa (n = 18)	19	1	18	20	8.07	7	0.882 ± 0.039	0.0177 ± 0.0011	0.023 ± 0.006	1.518
Total (n = 157)	44	8	36		10.13	57	0.948 ± 0.010	0.0222 ± 0.0005	0.027 ± 0.006	0.429

**n**; number of isolates, **S; **Number of segregating (polymorphic/variable) sites; **K; **Average number of pairwise nucleotide differences, **H; **Number of haplotypes, **Hd; **Haplotype diversity; **π; **Observed average pairwise nucleotide diversity; **dN-dS; **rate of non-synonymous mutations minus rate of synonymous mutations; **D; **Tajima's D test statistics.

**Table 2:** Comparison of different estimates of recombination events between all five study areas

**Regions**	**R^a^**	**R^b^**	**Rm**
Assam (n = 28)	0.178	81	9
Orissa (n = 35)	0.118	53.7	11
A & N (n = 40)	0.057	26	7
UP (n = 36)	0.016	7.5	8
Goa (n = 18)	0.074	33.7	6

Total (n = 157)	0.123	56.2	12

**Note: **The R and Rm were estimated excluding the sites containing alignment gaps or those segregating for three nucleotides. The R was computed using R = 4Nr, where N is the population size and r is the recombination rate per sequence (per gene) n, number of isolates; **R**^a^, recombination parameter between adjacent sites; **R**^b^, recombination parameter for entire gene; **Rm**, minimum number of recombination events between adjacent sites

**Figure 2:**
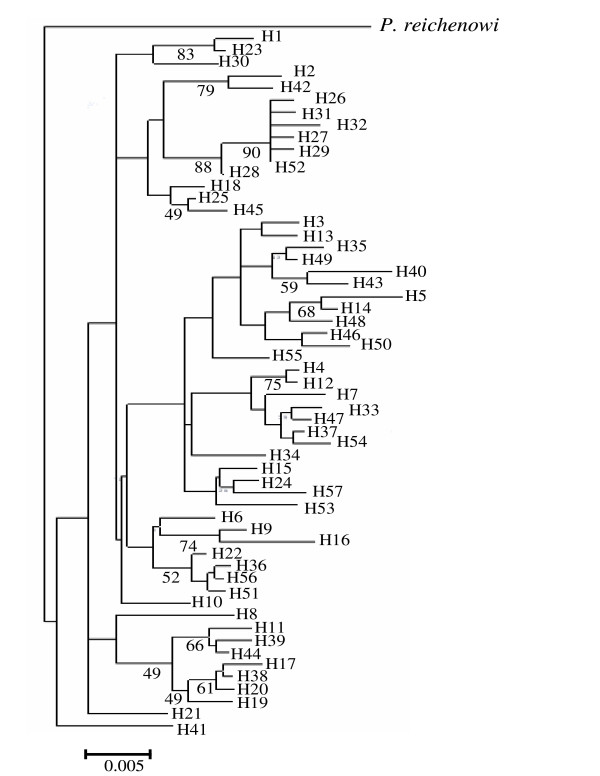
A neighbor-joining (NJ) tree depicting the relationships between different AMA1 haplotypes observed among Indian *P. falciparum *populations. The distance matrix was prepared using Kimura 2-parameter evolutionary model. Alignment substitutions were considered for analysis while gaps were ignored. Numbers below the line indicate percentage bootstrap values for 1000 replications. The scale bar represents a genetic distance. The partial AMA1 sequence of *P. reichenowi *[AJ252087], the closet species to *P. falciparum *was taken as an out group [35].

Inter-population nucleotide differences (Kxy) varied from 8.3 (between UP and Goa) to 11.53 (Assam and A & N) (Table 3). Similarly, the average number of nucleotide (Dxy) and net nucleotide (Da) substitutions per site between populations ranged from 0.018 to 0.025 and 0.00135 to 0.0755 respectively (Table 3). Although Fst statistics for the whole Indian *P. falciparum *populations was found to be 0.085, the Fst values between different geographical populations varied from 0.048 (between Orissa and Goa) to 0.133 (between UP and Orissa) (Table 3). The phylogenetic analysis of the sequences revealed that only 14 AMA1 alleles were shared by more than one geographical area (Additional File 2). Therefore, no region-wise clustering of the alleles was observed (Figure 2). Inter-population comparison of the total (TM) versus shared (SM) number of mutations also showed similar pattern of genetic differentiation as maximum number of shared mutations were found between Orissa & Goa (19 of 41 mutations were shared), and Goa and A & N (18 of 37 mutations were shared) (Table 3). Thus the data on phylogeny and Fst indicated moderate level of genetic differentiation and thus a limited movement of genes between populations.

**Table 3:** Inter-population genetic differentiation of the parasites

**S. No.**	**Population-1**	**Population-2**	**Kxy^a^**	**Dxy^b^**	**Da^c^**	**Fst^d^**	**TM/SM^e^**
1	Assam	UP	10.087	0.02212	0.00207	0.09339	38/26
2	Assam	Orissa	11.273	0.02472	0.00176	0.07101	43/35
3	Assam	Goa	10.764	0.02361	0.00271	0.11488	38/20
4	Assam	AN	11.531	0.02529	0.00191	0.07551	45/28
5	UP	Orissa	9.977	0.02188	0.00293	0.13380	41/25
6	UP	Goa	8.313	0.01823	0.00135	0.07421	26/20
7	UP	AN	9.909	0.02173	0.00237	0.10894	37/24
8	Orissa	Goa	9.481	0.02079	0.00100	0.04819	41/19
9	Orissa	AN	10.983	0.02409	0.00181	0.07517	47/28

10	Goa	AN	9.739	0.02136	0.00116	0.05414	37/18

^a^; Average number of nucleotide differences between populations

^b^; The average number of nucleotide substitutions per site between populations,

^c^; The number of net nucleotide substitutions per site between populations,

^d^; Fixation index, a measure of genetic differentiation between populations (range from 0 to +1),

^e^, TM, total number of mutations in both populations; SM, Numbers of mutations shared between populations

### Evidence of selection and recombination

The average difference of dN-dS for all 157 AMA1 sequences was 0.027 ± 0.006 SD, indicating that domain I is under positive natural selection (Table 1). The dN-dS differences were almost equal for Assam, Orissa and A & N populations but were greater than those observed for UP and Goa parasite populations (Table 1). The positive value 0.429 for Tajima's D also indicated that positive natural selection at domain I might be the reason for the increased allelic diversity observed (Table 1). Similar results were obtained when the McDonald & Kreitman (MK) test was applied to this data. The MK test showed more non-synonymous (dN) mutations within species (dN 36 vs. dS 3) and fixed difference between species (dN 7 vs. dS 4) indicating positive natural selection (Fisher's exact test P = 0.033, P > 0.01; Neutrality index = 6.85; α = -5.85).

**Figure 3:**
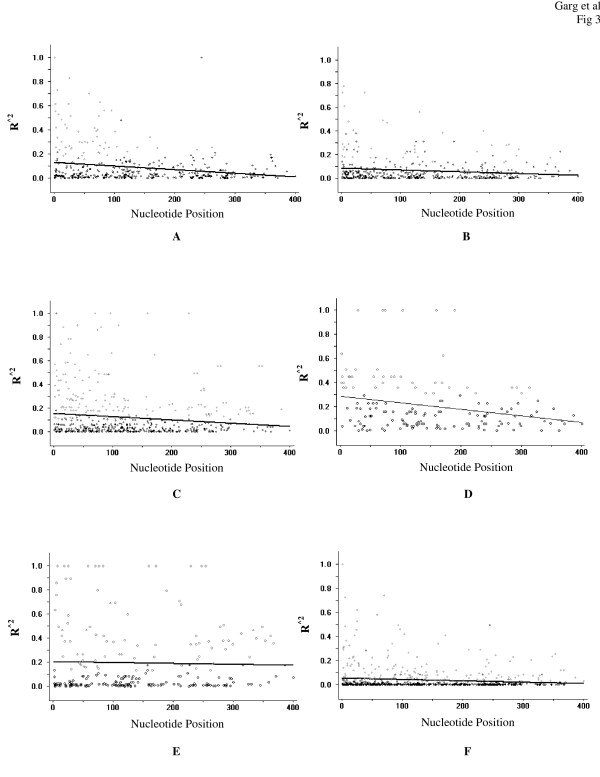
The linkage disequilibrium (LD) plot showing non-random association between nucleotide variants at different polymorphic sites. The R^^2 ^values are plotted against the nucleotide distances with two-tailed Fisher's exact test of significance using DnaSP. The value of LD index (range from -1 to +1) declines with increasing nucleotide distance, indicating that recombination events are taking place. The analysis was performed considering all polymorphic sites. (**A**) Assam, (**B**) Orissa, (**C**) A & N, (**D**) Goa, (**E**) UP, and (**F**) Total.

The minimum number of recombination events (Rm) between adjacent polymorphic sites for Assam, Orissa, A & N, UP and Goa isolates were 9, 11, 7, 8 and 6 respectively (Table 2). The value of R (both between adjacent sites and for the entire domain) was highest in Assam (0.178 and 81), followed by Orissa (0.118 and 53.7), Goa (0.0741 and 33.3), A & N (0.0571 and 26), and UP (0.0165 and 8) parasite population. The higher value of the recombination parameters (Rm and R) indicate that high meiotic recombination is taking place between the sites generating genetic diversity in the gene. As LD index R^^2 ^plotted against the nucleotide distances also demonstrated a decline across the entire 456 bp region (Figure 3), it can be assumed that intragenic recombination may be contributing to the increased diversity observed at AMA1 domain I.

## Discussion

Antigenic variation in the natural *P. falciparum *populations is one of the major obstacles in the development of an effective vaccine against malaria [1,29]. A vaccine based on one allelic form of an antigen might not provide full protective immunity against the heterologous *P. falciparum *strains, carrying other alleles of that antigen [30]. Therefore, multiple allelic forms of an antigen may need to be incorporated in a vaccine to make it more efficacious against any natural *P. falciparum *infection [13,31]. In this regard it becomes absolutely essential to investigate the antigenic repertoires of the *P. falciparum *populations from different malaria endemic regions. The complete AMA1 protein is comprised of three distinct domains [32]. This study includes only domain I as the rate of non-synonymous mutations at this domain has always been found to be higher due to the phenomenon of positive natural selection [13-16,28]. A total of 57 AMA1 haplotypes were observed from 157 *P. falciparum *isolates studied from five different regions of India. The H56 and H57 were the predominant haplotypes (Additional File 1). All measures of genetic polymorphisms showed regional variation among these five Indian *P. falciparum *populations (Table 1). The extent of genetic diversity depends on the malaria transmission intensity and the prevalence of malaria parasite in that region. The extent of genetic polymorphisms at domain I was higher amongst the isolates from Assam, Orissa and A & N as compared to those from UP and Goa isolates (Figure 1, Table 1). It is important to note that Assam, Orissa and A & N are considered as high malaria transmission areas whereas UP and Goa are low malaria transmission areas of India [33].

Analysis of the AMA1 sequences indicated that the level of diversity amongst Indian *P. falciparum *isolates was relatively lower than the reported diversity for African isolates but was quite similar to the Asian and South American populations [13,16,28]. A total of 35 AMA1 alleles (with π diversity 0.027 at domain I) were found when 51 Nigerian *P. falciparum *isolates were sequenced, while only 18 alleles (with π diversity 0.025 at domain I) were found when an almost equal number (n = 50) of isolates were sequenced from Thailand [16,28]. In another study, a total of 27 AMA1 alleles were found when 168 isolates (with π diversity 0.026 at domain I) were analysed from PNG [12]. It is important to note that number of polymorphic AMA1 codons among PNG [12] isolates (29 codons, 168 isolates) were similar to those observed amongst the Indian isolates (this study, 29 codons, 157 isolates). Although the number of haplotypes among Indian isolates (H = 57) were greater than those reported for the PNG isolates (H = 27) however, the π diversity was higher for PNG (π = 0.026) as compared to Indian isolates (π = 0.022).

Only 11 of the 57 haplotypes were identical to some of the AMA1 haplotypes observed in Thai, PNG and Nigerian *P. falciparum *isolates [12,13,16,28]. The H56 was the only haplotype which exhibited 100% identity with previously reported AMA1 alleles [AAG50135] from Indian isolate [13]. On the other hand 46 AMA1 haplotypes have been reported here for the first time.

Although polymorphisms were observed at 29 of the 152 codons of domain I, majority (17 of 29) of them were showing dimorphic amino acids which is in accordance with previous studies [12,13]. Only 5 alternative amino acid residues (G/D/H/Q/V) were present in the Indian parasite population at the highly polymorphic codon 197 of domain I as compared to 7 alternative substitutions reported from elsewhere [34]. A pdb structure of AMA1 using all these codon polymorphisms was also in accordance with previous studies [9]. Thus any vaccine developed using the AMA1 antigen will need to take into account all these variations observed within the gene.

The overall difference between dN and dS was positive (dN > dS) suggesting that domain I is under positive natural selection [25]. Similar results were obtained when Tajima's D and McDonald & Kreitman (MK) test were applied. Enormous evidence now supports the theory that the high allelic diversity at domain I is due to strong selection pressure by host immune response [16,17,25,34]. The estimates of inter-population comparisons (Kxy, Dxy, Da, TM/TS and Fst) supported moderate levels of genetic differentiation amongst the Indian populations which are relatively higher than the Fst values between Wosera & Nigerian (Fst = 0.0133), and Thai & Nigerian (Fst = 0.033) populations (Table 3) [12,13,28].

## Conclusion

In conclusion, the present study shows that domain I of AMA1 among Indian *P. falciparum *isolates exhibits fairly high allelic diversity that differs from one region to another. The regional differences in AMA1 allelic diversity may pose a problem for a vaccine based on this antigen. Results obtained in this study also demonstrate that polymorphisms at domain I is due to positive natural selection. Furthermore, moderate level of gene flow was observed amongst these parasite populations. This data on AMA1 polymorphism from Indian *P. falciparum *isolates will be valuable for the development of AMA1-based malaria vaccine.

## List of abbreviations

AMA1, apical membrane antigen; UP, Uttar Pradesh; A & N, Andaman and Nicobar Islands.

## Authors' contributions

SG performed PCR and sequencing. MTA analysed the sequences, performed statistical and phylogenetic analyses and drafted the manuscript. MKD, VD, AK, APD participated in the design of the study. YDS conceived of the study, and participated in its design and coordination, analysed the data and also drafted the manuscript. All authors read and approved the final manuscript.

## Supplementary Material

Additional file 1 Amino acid polymorphisms at domain I of the *P. falciparum *AMA1 among Indian isolates. The data provided represent the occurrence of codon polymorphisms at AMA1 domain I among Indian *P. falciparum *isolates. Click here for file

Additional file 2 Region-wise distribution and allelic frequencies of the AMA1 haplotypes. The data provided represent the region-wise distribution and frequencies of the AMA1 haplotypes found in the five study areas of India. Click here for file
