# Supplementary material for: Sequence diversity and natural selection at domain I of the apical membrane antigen 1 among Indian Plasmodium falciparum populations
Source: Malar J. 2007 Nov 22;6:154. doi: 10.1186/1475-2875-6-154 (PMC2211494; doi:10.1186/1475-2875-6-154)
Supplement: Additional file 1 — Amino acid polymorphisms at domain I of the P. falciparum AMA1 among Indian isolates. The data provided represent the occurrence of codon polymorphisms at AMA1 domain I among Indian P. falciparum isolates. [file 1475-2875-6-154-S1.pdf]

Table S1. Amino acid polymorphisms at domain I of the *P. falciparum* AMA1 antigen among Indian isolates (n = 157)

Haplotypes	No. of Isolates	Positions of codons and amino acid substitutions																												
		**162**	**167**	**172**	**173**	**175**	**187**	**189**	**190**	**196**	**197**	**199**	**200**	**201**	**204**	**206**	**207**	**218**	**225**	**228**	**230**	**242**	**243**	**244**	**267**	**269**	**282**	**283**	**285**	**296**
3D7*	1	N	T	G	N	Y	E	L	M	D	E	R	H	F	D	K	Y	S	I	N	K	D	K	D	E	K	I	S	Q	D
H1	1	-	K	-	-	D	N	-	I	-	D	-	R	V	-	E	-	-	N	-	-	Y	E	-	Q	-	K	L	-	-
H2	1	-	-	V	-	D	K	-	-	-	D	-	L	L	-	E	D	-	-	-	-	-	-	-	-	-	K	L	E	-
H3	1	-	-	-	-	D	N	-	-	N	D	-	D	-	N	E	-	-	N	-	E	Y	E	-	Q	-	K	L	-	-
H4	1	-	-	-	-	D	-	-	I	-	D	-	D	-	N	E	-	-	N	-	E	Y	-	-	Q	-	K	L	E	H
H5	1	K	-	-	K	D	N	-	I	N	G	K	D	-	-	E	D	-	N	K	E	Y	-	-	-	-	K	-	-	-
H6	1	K	-	E	-	D	K	-	-	-	D	-	D	-	-	E	-	-	N	-	-	Y	-	-	Q	-	K	-	-	-
H7	1	-	-	-	K	D	-	-	I	-	D	-	D	-	N	E	-	-	N	-	Q	Y	E	-	Q	-	K	L	E	-
H8	1	-	-	E	K	D	N	-	-	-	Q	-	-	-	-	E	-	-	N	-	E	Y	-	-	-	-	-	-	-	H
H9	1	K	-	E	-	D	-	-	I	-	D	-	D	S	-	E	-	-	N	-	E	Y	-	-	-	-	K	-	-	-
H10	1	-	-	E	-	D	D	-	-	N	D	-	-	-	-	E	-	-	N	-	-	Y	N	-	Q	-	K	-	-	-
H11	1	-	-	E	-	D	-	-	-	-	Q	-	-	-	-	-	-	-	-	-	-	-	-	-	Q	I	-	-	-	-
H12	1	-	-	-	-	D	K	-	I	-	D	-	D	-	N	E	-	-	N	-	E	Y	-	-	Q	-	K	L	E	H
H13	1	-	-	-	-	D	D	-	-	N	G	-	D	-	N	E	-	-	N	-	E	Y	-	-	Q	-	K	L	-	-
H14	1	-	-	-	K	D	N	-	I	N	G	K	D	-	-	E	-	-	N	-	E	Y	-	-	Q	-	K	-	-	-
H15	1	-	-	-	-	D	N	-	-	N	G	-	D	-	N	E	-	-	N	-	-	Y	N	-	Q	-	K	-	-	-
H16	1	K	-	E	-	D	-	-	I	-	D	-	D	S	-	E	-	-	N	-	Q	Y	E	-	Q	-	N	P	-	-
H17	1	-	-	E	-	D	N	P	-	-	Q	-	-	-	-	-	-	-	-	-	-	-	-	-	-	-	K	L	E	-
H18	1	-	-	E	-	D	-	-	-	-	D	-	R	-	-	E	-	-	N	-	-	Y	N	-	-	-	K	-	-	-
H19	1	-	-	E	-	D	K	-	-	-	Q	-	-	-	-	-	-	-	-	-	-	Y	E	-	Q	-	K	L	E	-
H20	1	-	-	E	-	D	-	-	-	-	Q	-	-	-	-	-	-	-	-	-	-	-	-	-	-	-	K	L	E	-
H21	1	-	-	-	-	D	-	-	-	-	D	-	-	-	-	E	-	-	-	-	-	-	-	-	-	-	-	-	-	-
H22	1	-	-	E	-	D	-	-	-	-	H	-	D	-	-	E	-	-	N	-	-	Y	N	-	-	-	K	-	-	-
H23	1	-	-	-	-	D	N	-	-	-	D	-	R	V	-	E	-	-	N	-	-	Y	E	-	Q	-	K	L	-	-
H24	1	-	-	-	-	D	N	-	-	-	G	-	D	-	-	E	-	-	N	-	-	Y	N	-	Q	-	K	L	E	-
H25	1	-	-	E	-	D	K	-	-	-	D	-	R	-	-	E	-	-	N	-	-	Y	N	-	Q	-	K	-	-	-
H26	1	-	-	-	-	-	-	-	-	-	D	-	L	L	G	E	D	-	N	-	-	Y	N	-	Q	-	K	-	-	-

H27	1	-	-	-	-	-	-	-	-	-	D	-	L	L	-	E	D	-	N	-	-	Y	N	-	Q	-	K	-	R	-
H28	1	-	-	-	-	D	-	-	-	-	D	-	L	L	-	E	-	-	N	-	-	Y	N	-	Q	-	K	-	-	-
H29	1	-	-	-	-	-	-	-	-	-	D	-	L	L	-	E	D	P	N	-	-	Y	N	-	Q	-	K	-	-	-
H30	1	-	-	-	-	D	N	-	-	-	D	-	R	-	-	E	-	-	-	-	-	-	-	-	Q	-	K	L	E	-
H31	1	-	-	-	-	-	-	-	-	-	D	-	L	L	-	E	V	-	N	-	-	Y	N	-	Q	-	K	-	-	-
H32	2	-	-	-	-	-	-	-	-	-	D	-	L	L	-	E	D	-	N	-	-	Y	N	-	Q	-	K	-	-	-
H33	2	-	K	-	-	D	-	-	I	-	D	-	D	-	N	E	-	-	N	-	Q	Y	E	-	Q	-	K	-	-	H
H34	2	-	-	-	-	D	K	-	-	-	G	-	D	-	N	E	-	-	N	-	-	-	-	-	-	-	K	L	E	-
H35	2	K	-	E	-	D	N	-	-	N	G	-	D	-	N	E	-	-	N	-	E	-	-	-	Q	-	K	-	-	-
H36	2	K	-	E	-	D	K	-	-	-	H	-	D	-	-	E	-	-	N	-	-	Y	N	-	-	-	K	L	-	-
H37	2	-	K	-	-	D	-	-	I	-	D	-	D	-	N	E	-	-	N	-	-	Y	E	-	Q	-	K	L	E	-
H38	2	-	-	E	-	D	K	-	-	-	Q	-	-	-	-	-	-	-	-	-	-	-	-	-	-	-	K	L	E	-
H39	2	K	-	E	-	D	-	-	-	-	Q	-	-	-	-	-	-	-	-	-	-	-	-	-	-	-	-	-	-	-
H40	2	-	-	-	-	D	N	P	-	N	G	-	D	L	N	E	-	-	-	-	-	-	-	-	Q	I	-	-	-	-
H41	2	-	K	-	-	D	N	-	I	-	Q	-	-	-	-	E	D	-	N	-	-	-	-	N	-	-	K	-	-	-
H42	2	-	-	E	-	D	K	-	-	-	D	-	L	L	-	E	D	-	-	-	-	-	-	-	Q	-	K	L	-	H
H43	3	-	-	-	-	D	N	P	-	N	G	-	D	L	N	E	-	-	N	-	E	-	-	-	-	-	K	-	-	-
H44	3	-	-	E	-	D	K	-	-	-	Q	-	-	-	-	-	-	-	-	-	-	-	-	-	-	-	-	-	-	-
H45	3	-	-	E	-	D	K	-	-	-	Q	-	R	-	-	E	-	-	N	-	-	Y	N	-	Q	-	K	-	-	-
H46	3	-	K	-	-	D	N	-	I	N	G	-	D	-	N	E	-	-	N	-	E	-	-	-	-	-	K	-	-	-
H47	3	-	K	-	-	D	-	-	I	-	D	-	D	-	N	E	-	-	N	-	-	Y	E	-	Q	-	K	L	-	H
H48	4	-	-	-	K	D	N	-	I	N	G	-	D	-	N	E	-	-	N	-	E	-	-	-	-	-	K	-	-	-
H49	5	-	-	-	-	D	N	-	-	N	G	-	D	-	N	E	-	-	N	-	E	-	-	-	Q	-	K	-	-	-
H50	5	-	K	-	-	D	-	-	I	N	G	-	D	-	N	E	-	-	N	K	E	-	-	-	-	-	K	-	-	-
H51	5	K	-	E	-	D	-	-	-	-	H	-	D	-	-	E	-	-	N	-	-	Y	N	-	-	-	K	-	-	-
H52	5	-	-	-	-	-	-	-	-	-	D	-	L	L	-	E	D	-	N	-	-	Y	N	-	Q	-	K	-	-	-
H53	5	-	K	-	-	D	N	-	I	N	G	-	D	-	-	E	D	-	N	-	-	Y	N	-	-	-	K	-	-	-
H54	6	-	K	-	-	D	-	-	I	-	D	-	D	-	N	E	-	-	N	-	-	Y	E	-	-	-	K	L	E	H
H55	8	-	-	-	-	D	-	-	-	N	V	-	D	-	N	E	-	-	N	-	-	-	-	-	Q	-	K	-	-	-
H56	21	K	-	E	-	D	K	-	-	-	H	-	D	-	-	E	-	-	N	-	-	Y	N	-	-	-	K	-	-	-
H57	25	-	K	-	-	D	N	-	I	N	G	-	D	-	N	E	-	-	N	-	-	Y	N	-	Q	-	K	L	E	-

*Accession numbers XP_001348015; AAN35928, AAB36701, n; number of isolates
