# Supplementary material for: Sequence diversity and natural selection at domain I of the apical membrane antigen 1 among Indian Plasmodium falciparum populations
Source: Malar J. 2007 Nov 22;6:154. doi: 10.1186/1475-2875-6-154 (PMC2211494; doi:10.1186/1475-2875-6-154)
Supplement: Additional file 2 — Region-wise distribution and allelic frequencies of the AMA1 haplotypes. The data provided represent the region-wise distribution and frequencies of the AMA1 haplotypes found in the five study areas of India. [file 1475-2875-6-154-S2.pdf]

Table S2. **Region-wise distribution and allelic frequencies of the AMA1 haplotypes**

Haplotypes[a]	Number (allelic frequency) of *P. falciparum* isolates				
	Assam (n = 28)	Orissa (n = 35)	A & N (n = 40)	UP (n = 36)	Goa (n = 18)
**H1**	**-**	**1 (0.028)**	**-**	**-**	**-**
**H2**	**-**	**1 (0.028)**	**-**	**-**	**-**
**H3**	**-**	**1 (0.028)**	**-**	**-**	**-**
**H4**	**-**	**1 (0.028)**	**-**	**-**	**-**
**H5**	**-**	**1 (0.028)**	**-**	**-**	**-**
**H6**	**-**	**1 (0.028)**	**-**	**-**	**-**
**H7**	**-**	**1 (0.028)**	**-**	**-**	**-**
**H8**	**-**	**1 (0.028)**	**-**	**-**	**-**
**H9**	**-**	**1 (0.028)**	**-**	**-**	**-**
**H10**	**-**	**1 (0.028)**	**-**	**-**	**-**
**H11**	**-**	**1 (0.028)**	**-**	**-**	**-**
**H12**	**-**	**1 (0.028)**	**-**	**-**	**-**
**H13**	**-**	**1 (0.028)**	**-**	**-**	**-**
**H14**	**-**	**1 (0.028)**	**-**	**-**	**-**
**H15**	**-**	**1 (0.028)**	**-**	**-**	**-**
**H16**	**1 (0.035)**	**-**	**-**	**-**	**-**
**H17**	**1 (0.035)**	**-**	**-**	**-**	**-**
**H18**	**1 (0.035)**	**-**	**-**	**-**	**-**
**H19**	**1 (0.035)**	**-**	**-**	**-**	**-**
**H20**	**1 (0.035)**	**-**	**-**	**-**	**-**
**H21**	**1 (0.035)**	**-**	**-**	**-**	**-**
**H22**	**1 (0.035)**	**-**	**-**	**-**	**-**
**H23**	**1 (0.035)**	**-**	**-**	**-**	**-**
**H24**	**1 (0.035)**	**-**	**-**	**-**	**-**
**H25**	**-**	**-**	**1 (0.025)**	**-**	**-**
**H26**	**-**	**-**	**1 (0.025)**	**-**	**-**
**H27**	**-**	**-**	**1 (0.025)**	**-**	**-**
**H28**	**-**	**-**	**1 (0.025)**	**-**	**-**
**H29**	**-**	**-**	**1 (0.025)**	**-**	**-**
**H30**	**-**	**-**	**1 (0.025)**	**-**	**-**
**H31**	**-**	**-**	**1 (0.025)**	**-**	**-**
**H32**	**-**	**-**	**2 (0.050)**	**-**	**-**
**H33**	**-**	**-**	**-**	**1 (0.027)**	**1 (0.055)**
**H34**	**-**	**-**	**-**	**2 (0.055)**	**-**
**H35**	**-**	**-**	**-**	**2 (0.055)**	**-**
**H36**	**-**	**-**	**-**	**-**	**2 (0.111)**
**H37**	**2 (0.071)**	**-**	**-**	**-**	**-**
**H38**	**2 (0.071)**	**-**	**-**	**-**	**-**
**H39**	**2 (0.071)**	**-**	**-**	**-**	**-**
**H40**	**-**	**2 (0.057)**	**-**	**-**	**-**
**H41**	**1 (0.035)**	**1 (0.028)**	**-**	**-**	**-**

Table S2 Contd.

H42	1 (0.035)	1 (0.028)	-	-	-
H43	2 (0.071)	1 (0.028)	-	-	-
H44	-	-	2 (0.050)	1 (0.027)	-
H45	-	-	3 (0.075)	-	-
H46	-	-	3 (0.075)	-	-
H47	-	-	-	1 (0.027)	2 (0.111)
H48	4 (0.142)	-	-	-	-
H49	-	4 (0.114)	1 (0.025)	-	-
H50	1 (0.035)	3 (0.085)	1 (0.025)	-	-
H51	2 (0.071)	-	-	1 (0.027)	2 (0.111)
H52	-	-	5 (0.125)	-	-
H53	-	-	-	1 (0.027)	4 (0.222)
H54	1 (0.035)	-	5 (0.125)	-	-
H55	1 (0.035)	-	-	4 (0.111)	3 (0.166)
H56	-	1 (0.028)	3 (0.075)	17 (0.472)	-
H57	-	7 (0.200)	8 (0.200)	6 (0.166)	4 (0.222)

n; number of isolates; [a], Haplotypes are same as defined in Table S1
